# Pilot Dietary Intervention with Heat-Stabilized Rice Bran Modulates Stool Microbiota and Metabolites in Healthy Adults

**DOI:** 10.3390/nu7021282

**Published:** 2015-02-16

**Authors:** Amy M. Sheflin, Erica C. Borresen, Melissa J. Wdowik, Sangeeta Rao, Regina J. Brown, Adam L. Heuberger, Corey D. Broeckling, Tiffany L. Weir, Elizabeth P. Ryan

**Affiliations:** 1 Department of Food Science and Human Nutrition, Colorado State University, Fort Collins, CO 80523, USA; E-Mails: amymarie@mac.com (A.M.S.); melissa.wdowik@colostate.edu (M.J.W.); 2 Department of Environmental and Radiological Health Sciences, Colorado State University, Fort Collins, CO 80523, USA; E-Mail: Erica.Borresen@colostate.edu; 3 Department of Clinical Sciences, Animal Population Health Institute, Colorado State University, Fort Collins, CO 80523, USA; E-Mail: Sangeeta.Rao@colostate.edu; 4 Cancer Center of the Rockies, University of Colorado Health, Fort Collins, CO 80522, USA; E-Mail: Regina.Brown@uchealth.org; 5 Department of Horticulture and Landscape Architecture, Colorado State University, Fort Collins, CO 80523, USA; E-Mail: Adam.Heuberger@colostate.edu; 6 Proteomics and Metabolomics Facility, Colorado State University, Fort Collins, CO 80523, USA; E-Mail: Corey.Broeckling@colostate.edu

**Keywords:** stool, microbiome, metabolome, microbial metabolites, dietary intervention, heat-stabilized rice bran

## Abstract

Heat-stabilized rice bran (SRB) has been shown to regulate blood lipids and glucose, modulate gut mucosal immunity and inhibit colorectal cancer in animal and human studies. However, SRB’s effects on gut microbial composition and metabolism and the resulting implications for health remain largely unknown. A pilot, randomized-controlled trial was developed to investigate the effects of eating 30 g/day SRB on the stool microbiome and metabolome. Seven healthy participants consumed a study meal and snack daily for 28 days. The microbiome and metabolome were characterized using 454 pyrosequencing and gas chromatography-mass spectrometry (GC-MS) at baseline, two and four weeks post-intervention. Increases in eight operational taxonomic units (OTUs), including three from *Bifidobacterium* and *Ruminococcus* genera, were observed after two and four weeks of SRB consumption (*p* < 0.01). Branched chain fatty acids, secondary bile acids and eleven other putative microbial metabolites were significantly elevated in the SRB group after four weeks. The largest metabolite change was a rice bran component, indole-2-carboxylic acid, which showed a mean 12% increase with SRB consumption. These data support the feasibility of dietary SRB intervention in adults and support that SRB consumption can affect gut microbial metabolism. These findings warrant future investigations of larger cohorts evaluating SRB’s effects on intestinal health.

## 1. Introduction

Heat-stabilized rice bran (SRB) is a nutrient-dense and phytochemical-rich food ingredient that is not widely consumed, but is gaining attention for its potential to help prevent multiple chronic diseases, including cardiovascular disease [1], type 2 diabetes [2,3], metabolic syndrome [1,4,5] and cancer [6,7,8]. Heat stabilization increases the shelf life of rice bran through inactivation of rancidity-inducing lipases and lipoxygenases, while retaining bioactivity [9,10]. The bioactive components of rice bran include, but are not limited to, γ-oryzanol, tocopherols, tocotrienols, phenolics (e.g., ferulic acid, caffeic acid), phytosterols (e.g., beta-sitosterol, cycloartenol) and specific free amino acids [6]. Whole grain brown rice reportedly changes the composition of gut bacterial phyla [11], increases anti-inflammatory SCFA levels in an *in vitro* canine microbiome model [12] and is a source of prebiotics [13]. Unidentified non-starch components of whole grains, including brown rice, were found to elicit changes in bacterial diversity, the *Firmicutes/Bacteroides* ratio and the bacterial abundance of the *Blautia* genus in the human gut microbiome [11]. In contrast to whole grain brown rice, SRB delivers a greater concentration of nutrients and phytochemicals to the gut and, as such, may differentially modulate stool microbiota and metabolites.

The structure and composition of the stool microbiome has shown profound associations with both human health and disease [14]. Emerging evidence supports the role of diet in modulating the structure of the gut microbiota [11,15,16]. Host diet, in turn, shapes not only the gut microbial composition, but also its metabolism [16]. Diet modifications have demonstrated effects on energy harvest, macronutrient metabolism and cancer risk, largely due to changes in microbially-produced metabolites that may promote or inhibit gastrointestinal health outcomes [17,18]. In particular, microbial metabolites have been found to exert pro- or anti-inflammatory activity on intestinal tissues and influence barrier function [19], host immune response [20,21], tumorigenesis [22,23] and tumor proliferation [24]. These metabolic perturbations result from changes in substrate availability, as well as diversity amongst microorganisms to ferment and biotransform specific dietary components. Dietary modulation of the microbiome and associated metabolism may prove a viable strategy for disease prevention and optimization of health.

The objective of this pilot dietary SRB intervention was to confirm the acceptability and feasibility of SRB supplementation in people and to assess the potential effects of the diet on the composition of the stool microbiome and metabolome. We hypothesized that SRB consumption would promote microbial changes and alter stool metabolite profiles. These alterations in stool microorganisms and metabolites may explain the benefits of SRB for intestinal health and account for the reported SRB bioactivities in preventing chronic disease. Findings from this pilot study provide compelling support and the rationale for larger cohort investigations of dietary SRB supplementation.

## 2. Materials and Methods

### 2.1. Pilot Trial Design and Participation

A four-week, pilot, randomized-controlled, single-blinded dietary SRB intervention study was completed in seven healthy adults with no history of cancer at Colorado State University (CSU) and is part of a community-based collaboration with the University of Colorado Health-North (UCH) in Fort Collins, CO, USA. Inclusion criteria for participants included no history of food allergies or major dietary restrictions, not currently taking cholesterol-lowering medications or non-steroidal anti-inflammatory drugs (NSAIDs), not currently pregnant or lactating, not a current smoker, no antibiotic use or probiotic use within the last month and no history of gallstones. The CSU Research Integrity and Compliance Review Office and the UCH-North Institutional Review Board approved this study protocol (CSU protocol #: 09-1520H, 02/18/2010, and UCH-North IRB #: 10-1038, 07/28/2010). Written informed consent was obtained from all participants prior to enrollment.

Participants received study meals and snacks that either included SRB (30 g/day) or that did not include SRB (control). Participants were instructed to consume one study-provided meal and snack each day and were not required to alter the remainder of their other daily food intake. To remain blinded to the intervention, the study-provided meals and snacks were labeled “Group A” or “Group C”. Participants self-recorded the amount of each study meal and snack consumed daily for compliance assessment. Participants also completed a 3-day food log (2 weekdays, one weekend day) each week to more accurately measure total dietary intakes. Food logs were entered and analyzed using Nutritionist Pro™ diet analysis software (Axxya Systems, Redmond, WA, USA), and each diet log analysis included average daily caloric intake, macronutrient, amino acid, vitamin and mineral profiles. Participants self-collected stool samples in coded specimen containers within 24 h of their scheduled study visit and provided the sample to the study coordinator at three required time points: baseline, 2 weeks and 4 weeks. Stool specimens were refrigerated by participants prior to arrival; DNA and SCFA aliquots were extracted within 24 h; and the remainder of the stool sample was stored at −20 °C until lyophilization and global metabolite extraction. All participants completed the trial without any reported adverse effects.

### 2.2. Nutritional Composition of SRB

#### 2.2.1. Heat Stabilization of Rice Bran

Rice bran was provided by the US Department of Agriculture-Agricultural Research Service (USDA-ARS) Dale Bumpers Rice Research Unit (Anna McClung) and was derived from a single source (*Oryza sativa* L. ssp. *japonica* var. Neptune). The rice bran was heat-stabilized by heating at 100 °C for three min to inactivate the lipase/lipoxygenase enzymes and prevent the bran from becoming rancid.

#### 2.2.2. Composition of SRB and Control Intervention Meals and Snacks

Seven meals (e.g., casseroles, soups) and six snacks (e.g., smoothies, granola, crackers) were developed by a registered dietitian and certified chef for both the absence and inclusion of SRB and covered a wide range of taste preferences. The placebo-control meals and snacks were similarly matched in their macronutrient content to the intervention and did not contain any SRB or brown rice. Similar palatability and appearance of both control and SRB meals and snacks were confirmed with a community taste test, including people with and without a history of cancer. These recipe and taste test trials were conducted in accordance with IRB-approved protocols (data not shown). Recipes for the dietary intervention meals and snacks were analyzed using NutritionistPro™ diet analysis software (Axxya Systems, Stafford, TX, USA). Each intervention meal and snack contained 15 g of SRB to achieve a total daily intake of 30 g.

### 2.3. Pyrosequencing of the Bacterial Community

#### 2.3.1. DNA Extraction, Amplification and Sequencing

Stool samples were subsampled three times with sterile cotton swabs. MoBio Powersoil DNA extraction kits (MoBio, Carlsbad, CA, USA) were utilized according to the manufacturer’s instructions to extract DNA. Sample DNA was stored at −20 °C prior to amplification steps. Sequencing libraries were prepared as described in [25]. Sequencing was conducted under contract by the University of South Carolina’s Engencore Sequencing Facility using a 454 Life Sciences GS FLX System with titanium chemistry.

#### 2.3.2. Analysis of Microbiota

All bacterial sequence read editing and processing was performed with mothur Ver. 1.28 [26] using the default settings, unless otherwise noted. The mothur software package is an open source bioinformatics tool used to analyze 16S rRNA gene sequences. Briefly, sequence reads were: (i) trimmed (options used with the trim command in mothur were as follows: bdiff = 1, pdiff = 2, qaverage = 30, minlength = 200, maxambig = 0, maxhomop = 8, flip = T); (ii) aligned to the bacterial-subset SILVA alignment [27]; (iii) filtered to remove vertical gaps; (iv) screened for potential chimeras using the uchime method; (v) classified using the Ribosomal Database Project’s naïve Bayesian classifier (RDP-NBC) training set for mothur [28]; all sequences identified as chloroplast were removed; (vi) sequences were screened (optimize = minlength-end, criteria = 95) and filtered (vertical = T, trump = . ), so that all sequences covered the same genetic space; and (vii) all sequences were pre-clustered (with up to two base-pair mismatches using the option diff = 2) to remove potential pyrosequencing noise and clustered into OTUs based on a 3% distance cutoff using the average-neighbor algorithm [29]. To remove the effect of sample size on community composition metrics, sub-samples of 450 reads were randomly selected from each stool sample. Sub-sampled community metrics were used to calculate alpha diversity community descriptors, including observed species richness (Sobs), Shannon’s diversity (H’) and evenness (E_H_) and Simpson’s diversity (S_D_) using the mothur implementation of these calculators. All sequence data are publicly available through the Sequence Read Archive (SRA) under study Accession Number PRJEB8075, which is available at [30].

### 2.4. Metabolite Profiling

#### 2.4.1. Metabolite Extraction and Detection by Gas Chromatography-Mass Spectrometry

Lyophilized stool metabolites were extracted using 80:20 MeOH:H_2_O and detected using GC-MS, as described previously [25]. For each sample, a matrix of molecular features defined by retention time and mass (*m/z*) was generated using XCMS software [31]. Features were normalized to total ion current, and the relative quantity of each molecular feature was determined by the mean area of the chromatographic peak among replicate injections (*n* = 2). Metabolites were identified by matching mass spectra to the National Institute for Standards and Technology (NIST 11) [32] and Golm metabolite databases [33] after deconvolution using AMDIS software [34].

#### 2.4.2. Short Chain Fatty Acid Determination

Frozen stool samples were extracted for short chain and branched chain fatty acids (SCFA and BCFA, respectively) using acidified water (pH 2.5), as described previously [25]. Peak areas were normalized to the total signal and represented as a percentage of total SCFA. Commercial standards of acetic acid, propionic acid, isobutyric acid, butyric acid, isovaleric acid, valeric acid, caproic acid and heptanoic acid (Sigma, St. Louis, MO, USA) were used to confirm compound identities.

### 2.5. Statistical Analysis and Data Visualization

The data on caloric intake, macronutrients and SCFA were checked for assumptions of normality. Due to the small sample size and non-normality, the SCFAs data were converted into ranks prior to performing a linear regression analysis. The “diet” groups and “time points” along with their interaction terms were used as factors to evaluate their impact on SCFA outcomes. Medians were used to describe the data. The analysis took into account the repeated measures on the same individual over time. A *p*-value of 0.05 was considered for determining statistical significance.

#### 2.5.1. Microbiota Analyses

The mothur implementation of the analysis of molecular variance (AMOVA) test, using a *p*-value threshold of 0.01, was applied to determine variation in community samples. AMOVA is a non-parametric version of the traditional analysis of variance (ANOVA) test that is widely used when testing genetic diversity. PCoA loadings were generated in mothur and visualized using R software (v3.0.1) [35]. The METASTATS [36] function within mothur was used to detect differentially abundant bacterial taxa in stool from SRB-consuming participants *versus* controls at a corrected *p*-value (expressed as the *q*-value and calculated based on a published algorithm [37]) threshold of 0.05.

#### 2.5.2. Metabolome Analyses

Stool metabolite features were identified via GC-MS. Differences in metabolite features between samples from baseline to 4 weeks in the SRB and control groups were determined using the Student’s *t*-test with a significance cutoff of <0.01. To minimize baseline inter-individual differences, metabolite data were first converted to the percent change from baseline that was calculated using the normalized area under the curve (AUC) as follows:

(4 week AUC − Baseline AUC)/(Baseline AUC) × 100 = % total

Negative values represent a decrease from baseline in a particular metabolite. In order to focus on metabolite changes due primarily to SRB intake rather than other food components that were common to the intervention meals and snacks, the mean change for each metabolite in the control group was subtracted from the mean change in the SRB group.

Individual SCFA concentrations were normalized as a percent of total measured SCFAs, and a weighted mean was calculated for each quantified compound. Statistical differences (*p* < 0.05) between stool samples from SRB-consuming participants and control participants were determined using a mixed model ANOVA representing a random effect and SRB intervention as a fixed effect (XLSTAT 2011.1, Addingsoft Corp, Paris, France).

## 3. Results

Completion of this pilot study demonstrated the feasibility of the placebo control, single-blinded dietary SRB intervention in healthy adults and established a standardized collection of stool samples for microbiome and metabolome assessment. All seven participants completed this pilot study between August 2010 and March 2011. Three participants were allocated to the control diet, and four participants were allocated to the SRB diet. Participant baseline characteristics are shown in Table 1.

### 3.1. Increased SRB Effects on Caloric and Macronutrient Intakes

**Figure 1:**
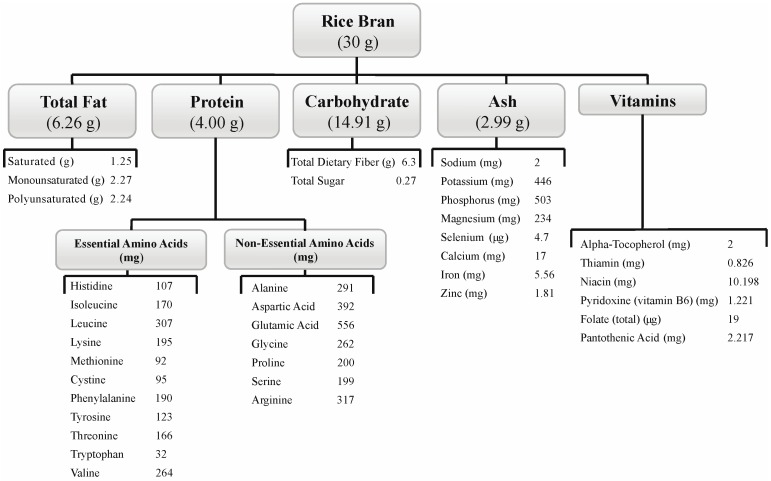
Nutrient composition of 30 g of heat-stabilized rice bran (SRB) [38]. The carbohydrate portion also includes unlisted starch.

Participants randomized to the SRB group consumed 30 g of SRB daily for the 28-day duration of the study, which compositionally included 6.26 g fat, 4.01 g protein, 14.91 g carbohydrate and a variety of vitamins and minerals (Figure 1) [38]. Even though all participants were free to consume brown rice or SRB outside of the intervention, the three-day food logs revealed that none of the participants from the control or SRB group were consuming whole grain brown rice during the study. The three-day food log analysis collected from participants each week revealed no significant change in caloric intake from Week 2 to Week 4 for either the control (*p* = 0.455) or SRB groups (*p* = 0.620). There was no significance between groups at the two-week or four-week time points (*p* = 0.966 and *p* = 0.394, respectively). Table 2 shows the caloric intakes for both groups. The median caloric intake for the SRB group was 1941 kcal at Week 2 and 1791 at Week 4. The control group had a median caloric intake of 2186 kcal at Week 2 and 2099 at Week 4. The control group had a significant decrease in protein intake at Week 4 compared to Week 2 (*p* = 0.001), and the SRB group had a significant decrease in protein intake at Week 4 compared to Week 2 (*p* < 0.0001). Carbohydrate intakes were not significantly different in the control group compared to the SRB group at Week 4 (*p* = 0.7). A similar pattern was shown for total fat intake at Week 4 (*p* = 0.99). The control group had a significant increase (*p* = 0.021) of fat intake at Week 4 compared to Week 2. The SRB group had significantly higher fiber intakes at Week 2 (*p* < 0.0001) when compared to the Week 2 control group, as well as at Week 4 compared to the control (*p* = 0.04).

**Table 1:** Baseline characteristics of study participants.

Characteristic	Control (*n* = 3)	Rice Bran (*n* = 4)
Age (years) ^a^	42.3 ± 21.7	42.8 ± 15.6
*Sex*		
Males (%)	2 (67%)	0 (0%)
Females (%)	1 (33%)	4 (100%)
BMI (kg/m^2^) ^a^	28.9 ± 6.9	22 ± 1.7
Total cholesterol ^a^ (mg/dL)	187 ± 57.2	197 ± 54.6
LDL ^a^ (mg/dL)	118 ± 50.3	127 ± 40.9
HDL ^a^ (mg/dL)	44 ± 12.6	54.3 ± 17.6
Triglycerides ^a^ (mg/dL)	125.7 ± 80.0	80 ± 35.0
*Fruit intake (X servings/day) ^b^*		
0 ≤ X ≤ 2	2	3
X > 2	1	1
*Vegetable intake (X servings/day) ^b^*		
0 ≤ X ≤ 2	1	1
X > 2	2	3
*Grain intake (X servings/day) ^b^*		
0 ≤ X ≤ 4	2	4
X > 4	1	0

^a^ Values are presented as the mean ± the standard deviation; ^b^ from the first three-day dietary food log.

**Table 2:** Total calories and macronutrient intake at the two-week and four-week time points for each study group *.

Dietary Intake	Control		Rice Bran	
	Week 2	Week 4	Week 2	Week 4
Calories (kcal)	2015.3 ± 325.0 (2186.4)	2047.8 ± 265.6 (2099.1)	2052.9 ± 410.3 (1940.6)	1925.3 ± 335.5 (1791.4)
Protein (g)	81.7 ± 13.7 (80.1)	77.6 ± 17.9 (77.6) ^b^	86.3 ± 14.0 (85.3)	68.9 ± 9.9 (71.1) ^b^
Carbohydrates (g)	264.6 ± 54.0 (290.8)	267.6 ± 53.0 (277.3) ^a^	253.0 ± 46.4 (243.9)	255.6 ± 58.3 (241.4) ^a^
Fat (g)	67.1 ± 13.9 (72.4)	74.6 ± 12.3 (81.0) ^b^	79.8 ± 16.6 (74.2)	75.4 ± 13.3 (74.3) ^a^
Fiber (g)	24.2 ± 3.0 (22.8) ^a^	23.5 ± 8.0 (19.4) ^b^	36.0 ± 7.5 (35.7) ^a^	32.4 ± 5.6 (31.9) ^b^

^*^ Values are presented as the mean ± SD (median). Medians are included, since ranks were compared in the analysis; ^a^ Significance (*p* ≤ 0.05) between the control and rice bran groups at time point; ^b^ Significance (*p* ≤ 0.05) at Week 4 compared to Week 2 for the specific diet.

### 3.2. Microbiome Changes with Consumption of SRB

**Figure 2:**
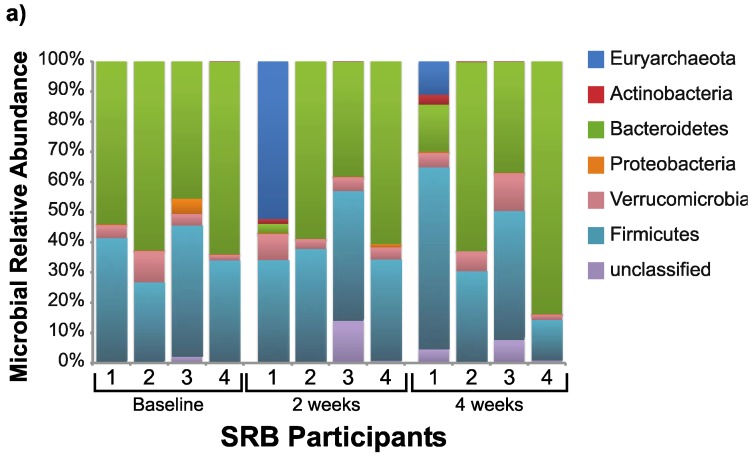

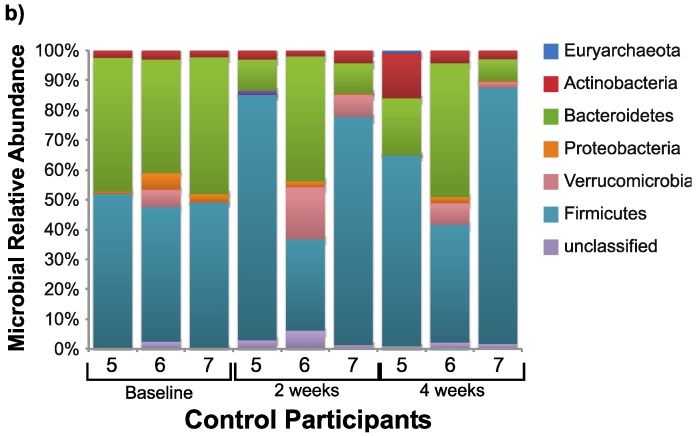
Phyla-level bacterial composition of stool samples for individual participants at baseline, two weeks and four weeks (**a**) with SRB and (**b**) without SRB.

On average, the coverage of the stool microbial community was 89% (Supplemental Table S1), and after subsampling, 2160 operational taxonomic units (OTUs) were detected in total. Stool bacterial richness, evenness and diversity remained constant during the SRB intervention with these healthy participants (Supplemental Table S1). The composition of the stool microbial communities at the phylum levels showed a high level of individual variation at both baseline and during the dietary intervention (Figure 2). Comparing bacterial composition at two and four weeks to the baseline at the phyla level revealed no significant changes in either SRB or control participants. After two weeks, eight OTUs belonging to the genera *Methanobrevibacter*, *Paraprevotella*, *Ruminococcus*, *Dialister*, *Anaerostipes* and *Barnesiella* showed significantly increased abundance (Table 3), and no OTUs showed reduced abundance with SRB. Additionally, increases in OTUs from the genera *Bifidobacterium* and *Clostridium* were noted at four weeks compared to the baseline. No significant changes at any taxonomic level were detected in stool bacterial composition for control participants.

**Table 3:** Percent change from baseline for bacterial operational taxonomic units (OTUs) that were significantly more abundant in the stool of individuals consuming SRB at two or four weeks.

Closest Hit in Database	2 weeks	*q*-Value	4 weeks	*q*-Value
*Methanobrevibacter smithii*	1201.00%	<0.001	210.73%	<0.001
*Paraprevotella clara*	352.87%	<0.001	156.71%	<0.001
*Ruminococcus flavefaciens*	128.49%	<0.001	79.02%	<0.001
*Dialister succinatiphilus*	86.59%	<0.001	57.47%	<0.001
*Bifidobacterium* sp.	2.79%	1.000	50.29%	0.003
*Clostridium glycolicum* (*Clostridium* cluster XI)	0.00%	1.000	40.71%	0.042
*Barnesiella intestinihominis*	277.35%	<0.001	66.31%	0.050
*Anaerostipes caccae*	90.09%	<0.001	69.63%	0.483
*Ruminococcus bromii*	66.77%	<0.001	29.47%	1.000

### 3.3. Metabolome Changes with Increased SRB

**Figure 3:**
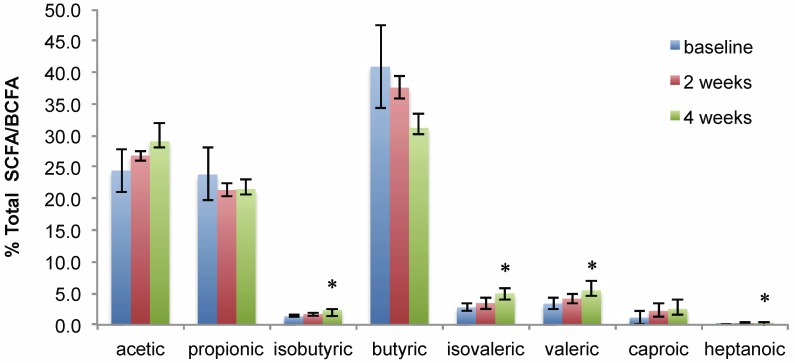
Proportional levels of SCFA and branched chain fatty acids (BCFA) in stool of SRB participants.

SCFAs, particularly acetate, propionate and butyrate, are primary products of carbohydrate fermentation [39]. Given that SRB’s macronutrient composition is 50% carbohydrate [10], SCFAs were quantified to assess changes driven by the SRB intervention. No significant increases in acetic, propionic, valeric, caproic and heptanoic acids were observed at two weeks or four weeks with the SRB intervention when compared to the baseline (Figure 3); however, butyric acid significantly decreased at Week 4 compared to the baseline and Week 2 (*p* = 0.025 and *p* = 0.0007, respectively). Furthermore, significant increases (*p* < 0.05) in isovaleric and isobutyric acid, both BCFAs, were observed at the two- and four-week rice bran intervention time points (Figure 3 and Supplemental Table S2).

Non-targeted metabolic profiling using GC-MS has been previously shown to identify stool metabolites that vary due to dietary modifications and demonstrated co-metabolic interactions between host and gut microbiota [40]. In order to focus on metabolite changes due primarily to SRB intake and not other foods included in intervention meals and snacks, the average change for each metabolite in the control group was subtracted from the changes in the SRB group. Despite this conservative approach, significant increases in abundance (as the mean peak area) for 28 stool metabolites (in addition to the increases in BCFA detailed above) were revealed at four weeks in the SRB group compared to the baseline. Furthermore, significant decreases were observed for eight additional stool metabolites. These metabolites function in pathways concerning synthesis, digestion and/or degradation of: amino acids, cholesterol and bile acids, phytochemicals and phenolics, lipids, vitamins and minerals and carbohydrates (Table 4). The metabolite with the largest increase was indole-2-carboxylic acid, a known phenolic component of whole grain rice [41], and this was increased approximately 12% at four weeks compared to the baseline (Table 4).

Significantly changed metabolites related to amine metabolic pathways were generally associated with purine and pyrimidine metabolism (Table 4). Decreases in stool leucine and glycine levels were observed in SRB participants, while BCFA metabolites of leucine, isoleucine and valine (Branched Chain Amino Acids) increased, as previously noted. Cholesterol and bile acid metabolites also increased with SRB (Table 4). Several beneficial phytochemicals increased with SRB, including two phytosterols known to be present in rice bran (Table 4) and the phenolics hydrocinnamic acid, benzoic acid and phenylacetic acid. Additionally, inositol phosphate was increased with SRB and is a potential product of phytic acid degradation, a known component of SRB. Metabolites in the lipid digestion and synthesis pathways also changed with SRB, including significant increases from the baseline for five saturated fatty acids (SFA) ranging from 1.0%–7.0 % (Table 4). Palmitic acid is a prominent component of SRB [42] and was found to be increased in stool following SRB consumption in this study (Table 4). Minor, but significant, increases were also observed for oleic acid and glycerol. Three fatty acids were seen to significantly decrease in stool after SRB intake: sebacic acid, 2-hexendioic acid and pentadecanoic acid.

**Table 4:** Candidate stool metabolites that differ between the baseline and four weeks in SRB participants (*p* ≤ 0.01). Known phytochemical and nutritional components of rice bran are marked with an asterisk (*).

Stool Metabolites	% change at 4 weeks	KEGG pathway
*Amino acids and nucleosides*		
Inosine	3.72%	Purine metabolism
Uridine	3.22%	Pyrimidine metabolism
Glutamic acid *	1.82%	Purine and pyrimidine metabolism
Glutaric acid	1.73%	Lysine degradation
Glycine *	−1.56%	Purine metabolism
Leucine *	−3.75%	Amino acid metabolism
*Cholesterol and bile acids*		
Cholest-8(14)-en-3-one	6.78%	N/A
Deoxycholic acid	2.69%	Secondary bile acid biosynthesis
Cholest5-en-3-ol-propionate	2.12%	N/A
Lithocholic acid	1.07%	Secondary bile acid biosynthesis
Cholesterol	0.51%	Steroid biosynthesis
*Phytochemicals and phenolics*		
Indole-2-carboxylic acid *	11.65%	N/A
Hydrocinnamic acid	4.31%	Phenylalanine metabolism
Alpha-tocopherol *	2.46%	Vitamin digestion and absorption
Benzoic acid	2.39%	Phenylalanine metabolism
Cycloartenol *	1.90%	Steroid biosynthesis
Pantothenic acid *	1.90%	Vitamin digestion and absorption
Phenylacetic acid	1.49%	Phenylalanine metabolism
Beta-sitosterol *	0.11%	Steroid biosynthesis
*Lipids*		
Myristic acid *	7.32%	Fatty acid biosynthesis
Caprylic acid	3.84%	Fatty acid biosynthesis
Lauric acid	3.03%	Fatty acid biosynthesis
Palmitic acid *	2.20%	Fatty acid biosynthesis
Stearic acid *	1.12%	Fatty acid biosynthesis
Azelaic acid	0.56%	N/A
Glycerol	0.55%	Galactose metabolism
Oleic acid *	0.15%	Fatty acid biosynthesis
Sebacic acid	−0.33%	N/A
2-Hexenedioic acid	−0.32%	N/A
Pentadecanoic acid	−1.90%	N/A
*Putative microbial metabolites*		
Indole-2-carboxylic acid *	11.65%	N/A
Hydrocinnamic acid ^a^	4.31%	Phenylalanine metabolism
Inositol monophosphate ^a^	3.90%	Inositol phosphate metabolism
Phosphoric acid ^a^	3.61%	Peptidoglycan synthesis
Deoxycholic acid	2.69%	Secondary bile acid biosynthesis
*Putative microbial metabolites*		
Benzoic acid ^a^	2.39%	Phenylalanine metabolism
Cycloartenol ^a^	1.90%	Steroid biosynthesis
Phenylacetic acid ^a^	1.49%	Phenylalanine metabolism
Stearic acid ^a^	1.12%	Fatty acid biosynthesis
Lithocholic acid	1.07%	Secondary bile acid biosynthesis
Beta-sitosterol ^a^	0.11%	Steroid biosynthesis
*Sugars ^b^*		
Maltose	−0.10%	Carbohydrate digestion
Ribose	−3.56%	Carbohydrate digestion
Glucose	−3.63%	Carbohydrate digestion

^a^ These metabolites may possibly be of plant origin, but can also be derived from microbial metabolism or modification of larger plant compounds, such as dietary fiber phenolics; ^b^ Sugar metabolites result from a wide range of metabolic pathways and could be of host, plant or microbial origin.

## 4. Discussion

The primary goal of this pilot dietary intervention was to confirm the feasibility of SRB consumption at 30 g/day in healthy adults and to determine if this amount was sufficient to induce detectable differences in stool microbiota and metabolites. Significant increases in eight OTUs were identified from human stool microbiota analysis at four weeks. There were nine stool metabolites increased and confirmed as SRB components and an additional eleven metabolite products of microbial metabolism that were elevated in stool at four weeks. The lack of adverse events and large-scale microbial community disruptions following the SRB intervention provide evidence for the feasibility of SRB consumption at 30 g/day for adults. Another goal of this pilot study was to set the stage with targeted microbial and metabolite endpoints that may influence intestinal health and colorectal cancer prevention outcomes. Results from this pilot study provided valuable insight into potentially important health-related changes that can be confirmed in future studies with larger sample sizes.

While there were no prior studies reporting the effect of SRB consumption on human microbiota, a human feeding study of 21 g/day of *Aspergillus*-fermented rice bran consumed for two weeks revealed no changes to the stool microbiota or SCFA profiles [43]. A dietary intervention using whole grain brown rice (60 g/day) for four weeks in human participants also produced no significant changes at the species level in participants’ gut microbiota or SCFA profiles [11]. In contrast, the present intervention detected changes in 6–8 OTUs at both two weeks and four weeks and significantly decreased butyric acid at four weeks. The differing outcomes may be explained by the amount (30 g/day) and rice fraction type (*i.e.*, SRB *vs.* whole grain), as compared to previous approaches. Confirming changes to bacterial species abundance and levels of branched and short chain fatty acids with SRB in a larger dietary intervention will be valuable*.*

BCFA levels increased in SRB participants’ stools and corroborate the 25% higher BCFA produced from rice compared to wheat, rye, corn and oats when subjected to *in vitro* digestion and fermentation using human stool samples [44]. The higher content of branched chain amino acids, valine and leucine, relative to other amino acids in SRB (Figure 1) may account for the significantly increased BCFA production (Table 4 and Supplemental Table S2) by gut commensals. In general, increased BCFAs are associated with diets high in animal fat and are consistent with protein degradation in the colon [45]. However, there is no direct evidence that BCFAs are causal of negative health outcomes. In fact, they may play a role in preventing human intestinal disease processes, as BCFAs were found in lower amounts in patients with irritable bowel syndrome (IBS) when compared to healthy controls [46]. Barrier function was investigated with human Caco-2 cells on a polycarbonate membrane, and transepithelial electrical resistance (TEER) was improved with BCFA-enriched culture media [47]. BCFAs may promote the establishment of beneficial gut bacteria, as short BCFAs are incorporated into longer BCFAs present in bacterial membranes. This process of BCFA-enrichment of bacterial membranes has been demonstrated *in vitro* for *Ruminococcus* [48]. Although *Bifidobacterium* spp*.* [49] also incorporate long BCFA in their membranes, similar studies on the incorporation of short BCFA have not been performed. BCFA production from SRB provides a novel mechanism by which SRB may improve intestinal health.

A recent *in vitro* study showed that hemicelluloses of SRB bind both cholesterol and bile acids [50]. Increased fecal extraction of bile acids was shown in rats fed SRB [51], and to our knowledge, this is the first evidence for elevated bile acid and cholesterol excretion in human stool following increased SRB consumption. Sequestration of bile acids and cholesterol is generally considered beneficial and a primary clinical and dietary means for lowering blood lipid profiles [52]. However, we did not expect to see any significant changes in the plasma lipid parameters of participants in this study, because we investigated a healthy population with serum lipids in normal ranges.

Several beneficial phytochemicals were significantly increased in the stools of SRB participants compared with the baseline (Table 4). These included two rice bran phytosterols: cycloartenol and beta-sitosterol. Cycloartenol has been shown in animal studies to significantly reduce blood cholesterol and triglycerides [53] and also reduced 12-O-tetradecanoylphorbol-13-acetate (TPA)-induced inflammation [54]. Additionally, in its trans-ferulate form, cycloartenol has anti-carcinogenic activity [55]. Beta-sitosterol scavenges free radicals and has shown potential as an anti-cancer drug through changed expression of beta-catenin and proliferating cell nuclear antigen (PCNA) [56]. Other increased phytochemicals include phenolics, namely hydrocinnamic acid, benzoic acid and phenylacetic acid. These compounds are all part of the phenylalanine metabolism pathway in KEGG [57]. Combinations of various derivatives of all three of these compounds in the form of fecal water extracts have previously been found to be anti-inflammatory by decreasing COX-2 activity [58]. While increased stool metabolites are not indicative of elevated systemic or host tissue levels of these compounds, they do have the potential to exert effects locally for the modulation of intestinal inflammation. The ability of this pilot intervention to detect significant increases in nine potentially SRB-derived metabolites (Table 4) underscores the potential for SRB intervention to modify the stool metabolome.

Of those metabolites significantly changed from the baseline, eleven are possibly of microbial origin (Table 4). The associated pathways for these metabolites suggest the following primary substrates, including: phenylalanine, primary bile acids and inositol phosphate. In addition, microorganisms produce beta-sitosterol and cycloartenol from a primary phytosterol gamma-oryzanol, which is known to be present in SRB [59]. Inositol phosphate can be produced by intestinal microbiota from diet-derived phytates, and previous research suggests this potential for phytate degradation [60]. The largest metabolite change noted in the current study was indole-2-carboxylic acid, which showed a mean 12% increase with SRB consumption. This indole derivative is a known component of brown rice, but indole derivatives are also microbial metabolites resulting from protein degradation, particularly the amino acid tryptophan [61]. In addition, diverse phenolic compounds associated with or bound to dietary fiber, such as ferulic and other hydroxycinnamic acids, are converted by gut microbiota to a few of the same metabolites [62,63]. These phenolic metabolites include phenylacetic and benzoic acids, which were significantly increased with SRB supplementation in the current study. Taken together, these findings suggest that microbial fermentation of SRB in the gut may shape the resulting metabolites and their metabolic activity.

## 5. Conclusions

Previous research efforts characterizing SRB have largely focused on nutrient contents, phytochemicals (e.g., antioxidants) and the effects of consumption for the prevention and management of major chronic diseases. However, SRB modulation of the ~100 trillion microorganisms in the human gastrointestinal tract and alteration of their metabolic activities may result in the production of chemicals that confer its reported bioactivity. Despite the expected high level of inter-individual variation in the microbiota and metabolome, this pilot study demonstrated that SRB intake of 30 g/day changes stool bacterial populations after two and four weeks and results in the significant alteration of multiple plant- and microbe-derived stool metabolites. Additionally, several target outcome measures for larger clinical trials with SRB were identified. For example, SRB-associated increases in BCFA production that may reduce gut permeability and encourage the growth of *Bifidobacterium* should be confirmed. Other metabolites targeted for quantification in future studies include colonocyte-feeding SCFA, microbiota-modulating secondary bile acids, anti-inflammatory SRB phytochemicals and indole-2-carboxylic acid as a candidate SRB intake biomarker in stool. This research emphasizes the value of pilot trials in confirming the feasibility and acceptability of the SRB intervention and targeting appropriate outcome measures prior to conducting research in a larger cohort. Understanding which organisms create bioactive SRB metabolites will be critical to achieving gastrointestinal disease prevention outcomes in people. Considering the microbial metabolism of SRB in humans will also improve our ability to advance its utility for improved intestinal health and the prevention of major chronic diseases.

## Supplementary Files

Supplementary File 1
