# Supplementary material for: Pilot Dietary Intervention with Heat-Stabilized Rice Bran Modulates Stool Microbiota and Metabolites in Healthy Adults
Source: Nutrients. 2015 Feb 16;7(2):1282–300. doi: 10.3390/nu7021282 (PMC4344588; doi:10.3390/nu7021282)
Supplement: Supplementary File 1 [file nutrients-07-01282-s001.docx]

Supplementary Information

**Table S1.** Stool bacterial diversity at baseline (time = 1) and four weeks (time = 3) during the intervention for both SRB and control participants. Nseqs = total number of sequences detected for that sample.

| **Group** | **Time** | **Nseqs** | **Coverage** | **S_obs_** | **S_D_** | **H’** | **E_H_** |
| --- | --- | --- | --- | --- | --- | --- | --- |
| SRB1 | 1 | 450 | 0.89 | 73 | 7.70 | 2.74 | 0.64 |
| SRB2 | 1 | 450 | 0.85 | 114 | 15.37 | 3.46 | 0.73 |
| SRB3 | 1 | 450 | 0.90 | 125 | 8.41 | 3.05 | 0.63 |
| SRB1 | 3 | 450 | 0.90 | 113 | 7.66 | 3.11 | 0.66 |
| SRB2 | 3 | 450 | 0.90 | 212 | 23.71 | 3.93 | 0.73 |
| SRB3 | 3 | 450 | 0.88 | 133 | 20.68 | 3.82 | 0.78 |
| *p*-value |  |  | 0.90 | 0.72 | 0.45 | 0.57 | 0.48 |
| Control 1 | 1 | 450 | 0.83 | 212 | 34.91 | 4.24 | 0.79 |
| Control 2 | 1 | 450 | 0.89 | 128 | 15.22 | 3.49 | 0.72 |
| Control 3 | 1 | 450 | 0.86 | 171 | 26.70 | 4.06 | 0.79 |
| Control 1 | 3 | 450 | 0.93 | 116 | 8.06 | 2.93 | 0.62 |
| Control 2 | 3 | 450 | 0.93 | 205 | 13.23 | 3.51 | 0.66 |
| Control 3 | 3 | 450 | 0.89 | 129 | 17.76 | 3.52 | 0.73 |
| *p*-value |  |  | 0.16 | 0.02 | 0.31 | 0.04 | 0.09 |

S_obs_ = total number of OTUs detected in a sample; SD = Inverse Simpson Index; H’ = Shannon Index;
E_H_ = Shannon Evenness Index.

**Table S2.** Short chain and branched chain fatty acids identified from targeted GC-MS analysis at baseline, two weeks and four weeks in SRB participants.

| **SCFA** | **Control (*n* = 3)** | | | **Rice Bran (*n* = 4)** | | |
| --- | --- | --- | --- | --- | --- | --- |
|  | **Baseline** | **Week 2** | **Week 4** | **Baseline** | **Week 2** | **Week 4** |
| Acetic Acid | 18.48 ± 1.82 (18.74) ^a,b,d^ | 24.98 ± 5.15 (26.58) ^a^ | 24.37 ± 2.55 (23.60) ^b^ | 24.46 ± 6.26 (24.74) ^d^ | 26.83 ± 2.28 (27.09) | 29.11 ± 5.34 (29.31) |
| Propionic Acid | 16.39 ± 2.42 (15.47) ^d^ | 15.63 ± 3.13 (14.54) ^d^ | 16.39 ± 3.75 (17.37) ^d^ | 23.83 ± 7.55 (23.32) ^d^ | 21.37 ± 1.30 (21.36) ^d^ | 21.54 ± 3.10 (20.28) ^d^ |
| Isobutyric Acid | 2.08 ± 0.57 (2.42) ^d^ | 1.98 ± 1.04 (1.99) | 1.42 ± 0.59  (1.06) ^d^ | 1.38 ± 0.41 (1.53) ^b,d^ | 1.71 ± 0.14 (1.72) ^c^ | 2.38 ± 0.41 (2.44) ^b,c,d^ |
| Butyric Acid | 44.98 ± 2.25 (44.29) | 43.25 ± 5.97 (41.68) | 45.36 ± 6.24 (43.83) ^d^ | 40.93 ± 12.17 (38.72) ^b^ | 37.57 ± 4.18 (39.03) ^c^ | 31.24 ± 4.60 (32.49) ^b,c,d^ |
| Isovaleric Acid | 5.51 ± 1.70 (6.10) ^d^ | 5.27 ± 3.64 (4.31) | 3.50 ± 1.97  (2.44) | 2.79 ± 1.08 (2.86) ^b,d^ | 3.44 ± 0.62 (3.33) ^c^ | 4.98 ± 1.78 (4.68) ^b,c^ |
| Valeric Acid | 6.25 ± 2.14 (7.00) ^d^ | 5.51 ± 2.80 (6.10) | 4.61 ± 2.11  (4.56) | 3.37 ± 1.59 (3.46)^d^ | 4.15 ± 1.81 (3.33) ^a^ | 5.47 ± 2.60 (4.68) |

**Table S2.** *Cont.*

| **SCFA** | **Control (*n* = 3)** | | | **Rice Bran (*n* = 4)** | | |
| --- | --- | --- | --- | --- | --- | --- |
|  | **Baseline** | **Week 2** | **Week 4** | **Baseline** | **Week 2** | **Week 4** |
| Caproic Acid | 5.78 ± 3.77 (7.64) | 2.92 ± 1.93 (3.48) | 4.06 ± 2.89  (4.53) | 2.32 ± 2.27 (2.12) | 2.99 ± 1.97 (3.60) | 3.41 ± 2.23 (4.34) |
| Heptanoic Acid | 0.54 ± 0.30 (0.43) ^d^ | 0.45 ± 0.42 (0.25) | 0.29 ± 0.24  (0.23) | 0.27 ± 0.23 (0.21) ^d^ | 0.50 ± 0.34 (0.31) | 0.55 ± 0.32 (0.55) |

^*^ Values are presented as the mean percentage of total SCFAs ± SD (median). Medians are included, since ranks were compared in the analysis; ^a^ Significance (*p* ≤ 0.05) between baseline and Week 2 of the diet group; ^b^ Significance (*p* ≤ 0.05) between baseline and Week 4 of the diet group; ^c^ Significance (*p* ≤ 0.05) between Week 2 and Week 4 of the diet group;
^d^ Significance (*p* ≤ 0.05) between control and rice bran at the time point.

© 2015 by the authors; licensee MDPI, Basel, Switzerland. This article is an open access article distributed under the terms and conditions of the Creative Commons Attribution license (http://creativecommons.org/licenses/by/4.0/).
